# Literature-based Resurrection of Neglected Medical Discoveries

**DOI:** 10.5210/disco.v6i0.3515

**Published:** 2011-04-20

**Authors:** Don R Swanson

**Affiliations:** 1 Division of the Humanities, University of Chicago, Chicago, ILUnited States

## Abstract

It is possible to find in the medical literature many articles that have been neglected or ignored, in some cases for many years, but which are worth bringing to light because they report unusual findings that may be of current scientific interest. Resurrecting previously published but neglected hypotheses that have merit might be overlooked because it would seem to lack the novelty of "discovery" -- but the potential value of so doing is hardly arguable. Finding neglected hypotheses may be not only of great practical value, but also affords the opportunity to study the structure of such hypotheses in the hope of illuminating the more general problem of hypothesis generation.

## Editor’s Note

This article, while incomplete, is noteworthy both in a historical and in a technical sense. Historical, because it sheds further light on the magnesium-migraine connection which is one of the examples that launched the field of literature-based discovery. Technical, because it draws attention to an important, and still poorly understood problem in scientometrics – how to identify findings in the literature that were neglected at the time of their publication but that are important and deserve to be “rehabilitated.” The article is being published in DISCO with the permission of Don, whose poor health precludes him from finishing it himself. Several notes have been added at the end of the article to provide open peer review and commentary. I invite readers to add their own comments online. (To add comments, go to the journal website . When viewing the abstract or PDF online, there is a link to the right of the page; note that registration is required in order to add comments.) 

## Introduction

It is possible to find in the medical literature many articles that have been neglected or ignored, in some cases for many years, but which are worth bringing to light because they report unusual findings that may be of current scientific interest. An example will introduce my story about how to identify and characterize these articles. The following abbreviated Medline record represents a neglected article, later resurrected, that reports a medical discovery:

PubMed ID: 4725298

Author:    Vosgerau, H.

Title:     Zur Behandlung der Migrane mit Magnesiumglutamat.   [Migraine therapy with magnesium glutamate.]

Source:    Therapie der Gegenwart.  112(4): 640 passim. 1973 Apr.

Vosgerau reported consistent and rapid termination of migraine attacks in 10 patients who were treated with injections of magnesium glutamate. He was not the first to treat migraine with magnesium, but his report apparently was the first to become the main topic of a medical journal article. Earlier, Simon (1967, p.59) had claimed success in terminating migraine attacks with an injection of magnesium glutamate, but he wrote only six sentences about it, buried in a book on the pharmacology of magnesium.

A search of the Science Citation Index showed that during the next fifteen years after its publication Vosgerau's article had been cited only twice. Moreover, a title search of Medline for "migraine AND magnesium" as late as 1987 yielded only the single record by Vosgerau, and a similar but broader search that included medical subject headings (MeSH) yielded only 4 records at that time. The absence of citations to an article over an extended period immediately following its publication, together with the scarcity of other works that investigate the same problem, are plausible markers for neglect.

Vosgerau's article contains, in the title, a biologically meaningful co-occurrence of a substance term and a disease term. To a human observer, it is clear from the word "therapy" in the title that the substance is expected to influence (beneficially) the course of the disease. The same implication is reflected in the complementary subheadings assigned to the MeSH terms for magnesium and migraine in the full Medline record for that article. "Therapeutic use" is attached to the heading "magnesium" and "drug therapy" is attached to the heading "migraine". The possible role of subheadings in limiting the search space for other neglected discoveries merits investigation. Evidence that subheadings may be valuable in a similar though not identical context is discussed elsewhere (Swanson et al, 2006).

Hence, instead of searching for "discoveries" in general, we propose to address, at least initially, the better defined and more manageable problem of searching for substances or external agents that can or might influence diseases. Granted that large and important areas of medicine, including genetics, are not necessarily included in that description, this problem nonetheless covers much ground. Just how much can be estimated from the scope of the Medical Subject Heading (MeSH) schedules. The 2002 MeSH tree structures yielded a count of 9284 C-terms (diseases) and 32726 D-terms (substances, including entry terms). To learn which substances have been investigated for their effects on which diseases one can search Medline for each disease- substance pair of interest. The search space of all possible pairs is impressively large -- over 300 million. At a later point, we report results of a small randomized search experiment.

The fact that the words "migraine" and "magnesium" both appear in the (translated) title of the Vosgerau paper should not be taken lightly. It signals that the author regarded the connection between migraine and magnesium as important enough to justify being the main topic of an entire article, a point perhaps not unrelated to whether a substance -disease term - co-occurrence is likely to be trivial or important, how readily the context of the relationship can be seen, and whether title-searching should be used to find other such articles. 

## On the question of resurrection

In 1988 I reported an analysis of eleven indirect or implicit connections between migraine and magnesium deficiency found by studying their separate literatures. Reports that directly connected magnesium with migraine were scarce. I discussed the few I had found, including those by Simon (1967) and by Vosgerau (1973) mentioned above, as well as papers by Altura (1985) and by Jain et al (1985). Although my purpose was primarily to bring to light possibly unnoticed implicit connections between migraine and magnesium, irrespective of any reports directly connecting them, it was secondarily to call attention to direct connections that may not have been appreciated as standing almost alone as a bridge between two otherwise unconnected literatures. (Swanson 1988, p. 546-547).

That my citing the few early papers reporting a direct magnesium-migraine connection may have awakened interest in them is suggested by the subsequent citation pattern. The number of citations to the above pre-1988 reports that I discussed was only 2 for Vosgerau and none for Altura or Jain, prior to 1988, but in the ensuing 10 years (1988-1998) there were about 57 citing articles -- 14 for Vosgerau, 28 for Altura, and 15 for Jain. All but 2 of these 57 also cited my above paper (Swanson 1988), thus supporting the possibility that it was indeed the latter that was responsible for awakening a dormant interest in the pre-1988 migraine-magnesium work, and so could be considered as an example of literature-based resurrection.

Just why the paper by Vosgerau had been apparently ignored for 15 years may not be knowable, but a plausible guess is that not enough was known in 1973 about the biological mechanisms or pathways by which magnesium could influence migraine. Perhaps calling attention in 1988 to the indirect evidence in support of 11 possible pathways was sufficient to trigger wider interest in the role of magnesium in migraine.

Once a direct connection that seems to be lonely and neglected is detected, then the work of resurrection, by the above argument becomes that of analyzing pathways or mechanisms that might not be known. For the express purpose of assisting such an analysis, the set of programs and processes called Arrowsmith was created. Arrowsmith has two modes of operation -- hypothesis generation, and hypothesis testing. The above analysis shows that finding "neglected discoveries" can contribute to hypothesis generation, and, by so doing, provide input to the Arrowsmith hypothesis-testing mode. The latter then can help find the intermediate mechanisms of biological action constituting pathways that might illuminate and ignite interest in the neglected discoveries.

## Arrowsmith as pathfinder; hypothesis-testing mode

The biological mechanisms or pathways by which agent A might influence disease C are called, collectively, B. Pathways A-B established within one set of articles and separate pathways B-C within a second, non-overlapping, set, suggest that bringing together these two types of pathway might reveal previously unknown A-B-C pathways worth investigating.

The "pathway problem" as stated above divides naturally into two sub-problems, depending on our state of knowledge about A, and assuming that a single specific disease C is given. 

If a single specific A is hypothesized at the outset, we define a "hypothesis-testing" sub-problem, and attempt to find all plausible pathways, B, given both A and C. In this sub-problem, a list of B-candidates is generated by computer from words, phrases, or index terms common to the A and C bibliographic records in a database such as Medline. To then recognize which among the candidates could plausibly be part of a biological pathway requires, in general, human expertise and judgment. If A is unknown at the outset, then we define "hypothesis generation" as the sub-problem of developing a list of plausible A-terms.

## The context of literature-based discovery

In our previous work on literature-based discovery, the case in which the A and C literatures were disjoint -- i.e. had no articles in common -- has been of central interest, for it implies that no direct A-C connection had been documented. Hence creating a novel though implicit A-C connection became the main objective -- to find undiscovered public knowledge (Swanson and Smalheiser, 1997).

In the present paper, attention is shifted to cases in which the A-C intersection is not a null set but a set which instead is populated by just few articles. Those few articles are very likely to contain the kind of hypotheses we are trying to generate, and may well have the additional advantage of reporting preliminary tests based on clinical or laboratory research, irrespective of whether the biological mechanisms or pathways have also been investigated or discovered. Resurrecting previously published but neglected hypotheses that have merit might be overlooked as an approach to hypothesis generation because it would seem to lack the novelty of "discovery" -- but the potential value of so doing is hardly arguable. The connection explosion within the literature is vast, and even useful stuff gets lost. Finding at least some of it supplements, but does not replace, the more challenging problem of generating hypotheses de novo. The neglected hypotheses found can be treated as input to the hypothesis-testing mode of Arrowsmith in order to identify possible pathways or mechanisms.

Finding neglected hypotheses may be not only of great practical value, but also affords the opportunity to study the structure of such hypotheses in the hope of illuminating the more general problem of hypothesis generation.

## A pilot test based on randomized searching

From 9284 C-terms extracted from MeSH, 100 terms were selected using a random number generator. Title searches derived from the selected MeSH terms were constructed. Some of the terms were then omitted, for several reasons (see MeSH stoplist discussion elsewhere (Swanson et al, 2006), including terms that led to either too many or too few postings in a title-search. 47 terms remained, and are shown in Table 1.

From 32726 D-category substance entry-terms, 30 were selected at random. Entry terms were matched to their corresponding MeSH terms, and a suitable title search was constructed. 12 terms were omitted in this process and the remaining 18 are shown in Table 2. To these random selections, "migraine" was added to the disease list and "magnesium" to the substance list, to provide a known case as a benchmark.

**Table 1:**
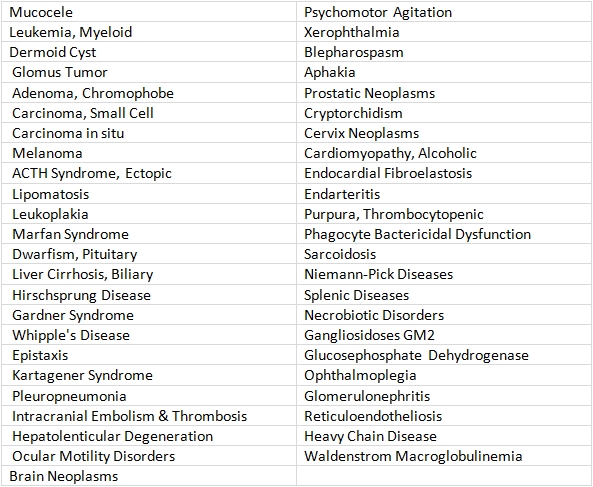
Disease terms: random selection – edited

**Table 2:**
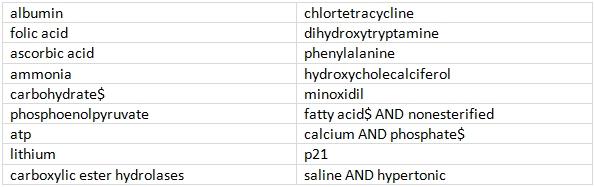
Substance terms: random selection -- edited

Using MEDLINE, and limiting all searches to the period 1965 to 1987, all 846 intersections of the 18 substances and 47 diseases were formed from a title search, narrowed down in some cases by using appropriate subject headings. There were 789 null and 57 non-null intersections; of the latter, 36 were small enough to qualify as "neglected" (5 or fewer articles -- based on a broader search than just titles, the threshold of 5 being somewhat arbitrary).

A search of the Science Citation Index (all years) was conducted for all articles corresponding to the 36 disease-substance intersections. Only those disease-substance articles that had 5 or fewer citations prior to 1988 were retained, of which there were 14. These 14 articles, which represented 9 disease-substance searches, fulfilled the two criteria for being considered as neglected.

Table 3 shows, for each of these 9 searches (14 articles) the number of citing references before and after 1988. The migraine and magnesium results are shown separately from the randomized group. The latter accounted for 5 of the 9 searches and 8 of the 14 articles. Table 4 lists authors and titles for the 14 articles.

**Table 3:**
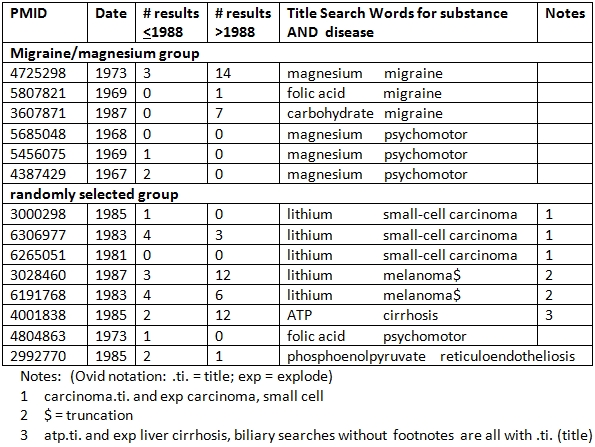
Citation count and search strategy

**Table 4:**
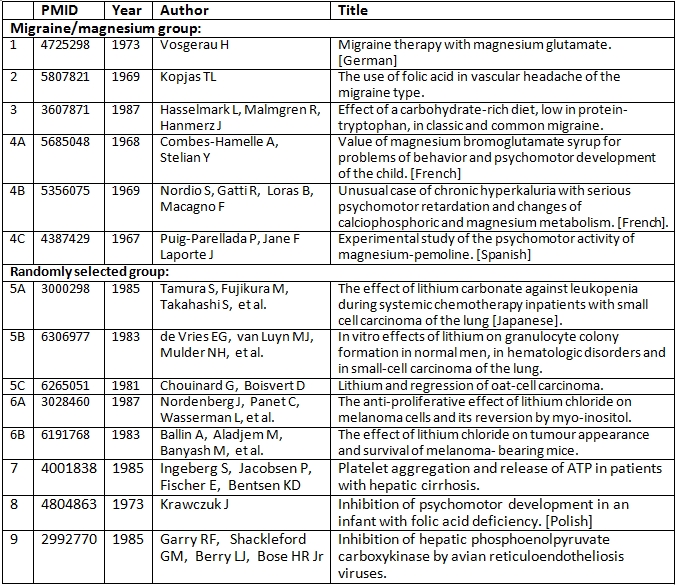
Authors and Titles

## Observation on subheadings

The titles of 11 of the 14 articles make clear to a human observer that they involve the influence of an externally administered agent on a disease, which fulfills the original intent of the search; the other three articles however (#4B, #7, and #9) do not directly fulfill that intent. The same inference can be drawn from the subheadings assigned to the substance and disease MeSH terms. Should a larger scale study bear out these results, it is possible that automatic filtering of the output based on pre-specified subheadings will prove to be of value.

## Addendum

The above reported experiment as it stands is incomplete; this draft reports work in progress. The next step is to repeat each of the final 9 substance-disease searches for the post 1988 time period. The purpose of conducting the above search as though the date were 1988 was to provide visibility on what happened after that date to these supposedly neglected discoveries. The next step after that is to conduct an Arrowsmith 2-node search using the same two terms as in the above 9 searches.

The purpose of the randomized approach was to see what would emerge in the way of "neglected discoveries" from a more or less mindless -- randomized -- combination of substances and diseases. Using this as a baseline makes it easier to evaluate more

intelligently-selected categories of the MeSH schedule for disease-substance combinations, an evaluation that may also shed light on the design of a menu of AA choices for the kiwi-server based 1-node search [Editor's note: This refers to Don's website at http://kiwi.uchicago.edu/, which is no longer being maintained.] Indeed it seems natural that the AA 1-node approach eventually will have built into it the "resurrection" approach presented here, simply because both are addressed to the problem of hypothesis generation. The role of the Science Citation Index at present isn't easily accomodated by Arrowsmith (http://arrowsmith.psych.uic.edu), but it is not yet clear that the SCI is indispensable for our purposes.

## References

[1] BM. Altura (1985). Calcium antagonist properties of magnesium: implications for antimigraine actions. Magnesium 4: 169-175, 1985. Magnesium.

[2] Ballin A, Aladjem M, Banyash M, Boichis H, Barzilay Z, Gal R, Witz I P (1983). The effect of lithium chloride on tumour appearance and survival of melanoma-bearing mice.. Br J Cancer.

[3] Chouinard G, Boisvert, D. (1981). Lithium and regression of oat-cell carcinoma.. Can Med Assoc J.

[4] Combes-Hamelle A, Stelian Y (1968). Intérêt du sirop de bromoglutamate de magnésium dans les troubles du comportement et le développement psycho-moteur de l'enfant. [Value of magnesium bromoglutamate syrup for problems of behavior and psychomotor development of the child]. Ann Pediatr (Paris).

[5] Vries E G, Luyn M J, Mulder N H, Bins M, Halie M R (1983). In vitro effects of lithium on granulocyte colony formation in normal men, in hematological disorders and in small-cell carcinoma of the lung.. Acta Haematol.

[6] Garry R F, Shackleford G M, Berry L J, Bose Jr, H.R. (1985). Inhibition of hepatic phosphoenolpyruvate carboxykinase by avian reticuloendotheliosis viruses.. Cancer Res.

[7] Ingeberg S, Jacobsen P, Fischer E, Bentsen K D (1985). Platelet aggregation and release of ATP in patients with hepatic cirrhosis.. Scand J Gastroenterol.

[8] Jain, AC, Sethi NC, Babbar PK (1985). A clinical electroencephalographic and trace element study with special reference to zinc copper and magnesium in serum and cerebrospinal fluid (CSF) in cases of migraine.. J. Neurol..

[9] Hasselmark L, Malmgren R, Hannerz J (1987). Effect of a carbohydrate-rich diet, low in protein-tryptophan, in classic and common migraine.. Cephalalgia.

[10] Kopjas T L (1969). The use of folic acid in vascular headache of the migraine type.. Headache.

[11] Krawczuk J (1973). Zahamowanie rozwoju psychoruchowego u niemowlecia zwiazane z niedoborem kwasu foliowego. [Inhibition of psychomotor development in an infant with folic acid deficiency. Pediatr Pol.

[12] Nordenberg J, Panet C, Wasserman L, Malik Z, Fuchs A, Stenzel K H, Novogrodsky, A. (1987). The anti-proliferative effect of lithium chloride on melanoma cells and its reversion by myo-inositol.. Br J Cancer.

[13] Nordio S, Gatti R, Loras B, Macagno F (1969). Propos d'une observation particulière d'hyperkaliurie chronique avec retard psychomoteur grave et altérations du métabolisme calciophosphorique et du magnésium. [Unusual case of chronic hyperkaluria with serious psychomotor retardation and changes of calciophosphoric and magnesium metabolism. Pediatrie.

[14] Puig-Parellada P, Jane F, Laporte J (1967). Estudio experimental de la acción psicomotora de la pemolina-magnesio. [Experimental study of the psychomotor activity of magnesium-pemoline. Rev Esp Fisiol.

[15] KH. Simon (1967). Magnesium. Physiologie: Pharmakologie: Klinik.

[16] DR. Swanson (1988). Migraine and magnesium: eleven neglected connections. Perspect. Biol. Med..

[17] Swanson D R, Smalheiser N R (1997). An interactive system for finding complementary literatures: a stimulus to scientific discovery. Artificial Intelligence.

[18] Swanson D R, Smalheiser N R (2006). Ranking indirect connections in literature-based discovery: The role of Medical Subject Headings (MeSH. JASIST.

[19] Tamura S, Fujikura M, Takahashi S, Maebo A, Nakano T, Yamada W, Fujii J, Nabeshima K, Hada T, Higashino K (1985). The Effect of Lithium Carbonate Against Leukopenia During Systemic Chemotherapy Inpatients with Small Cell Carcinoma of the Lung.. Gan To Kagaku Ryoho.

[20] Vosgerau H Ther Ggw (1973). Zur behandlung der migraine mit mangesiumsglutamat. [Migraine therapy with magnesium glutamate. Ther. Ggw.

